# Grandparenting and the Receipt of Filial Piety From Adult Children in Chinese American Families

**DOI:** 10.1093/geroni/igaa057.1110

**Published:** 2020-12-16

**Authors:** Jeung Hyun Kim

**Affiliations:** Syracuse University, Syracuse, New York, United States

## Abstract

The current study explores the association between grandparent caregiving by Chinese American elders and their perceived receipt of filial support from their adult children, called filial piety (xiao). Many studies find a correlation between grandparent caregiving and filial behaviors from their adult children, which is notably higher among minority families, especially among Asians than among white families, stimulated by the norm of reciprocity, familism, and extended kinship. Drawing from the theory of intergenerational relationships, social exchange theory, and the role theory, this study questions whether a more active engagement in grandparenting renders higher levels of filial piety returns from adult children. It uses the PINE data, a survey on the wellbeing of Chinese American elders in Chicago. The results show that more hours of grandparent caregiving relate to higher returns of filial piety perceived by older parents. Correspondingly, though with a marginal significance, more pressures to take care of a grandchild from adult children reduce the elders’ perception of filial piety receipt. No interaction effect is found between the grandparenting hours and the pressure from adult children. Additionally, Chinese American elders possessing higher levels of education, mastery, and longer stays in the US perceive lower levels of filial piety receipt from adult children. Discussion will focus on how grandparent caregiving can be mutually beneficial and strengthen intergenerational relationships among Chinese American families.

